# Ultrasound-guided fine-needle aspiration/biopsy-based pancreatic organoids establishment: an alternative model for basic and preclinical research

**DOI:** 10.1093/gastro/goad019

**Published:** 2023-04-10

**Authors:** Sheng Chen, Min Wang, Lei Liu, Guodong Wang, Lei Wang, Changqing Zhong, Chao Gao, Wei Wu, Lianyong Li

**Affiliations:** Department of Gastroenterology, PLA Strategic Support Force Medical Center, Beijing, P. R. China; State Environmental Protection Key Laboratory of Environmental Sense Organ Stress and Health, PLA Strategic Support Force Medical Center, Beijing, P. R. China; Department of Gastroenterology, PLA Strategic Support Force Medical Center, Beijing, P. R. China; Department of Gastroenterology, PLA Strategic Support Force Medical Center, Beijing, P. R. China; Department of Gastroenterology, PLA Strategic Support Force Medical Center, Beijing, P. R. China; Department of Medical Psychology, PLA Strategic Support Force Medical Center, Beijing, P. R. China; Department of Gastroenterology, PLA Strategic Support Force Medical Center, Beijing, P. R. China; Department of Gastroenterology, PLA Strategic Support Force Medical Center, Beijing, P. R. China; State Environmental Protection Key Laboratory of Environmental Sense Organ Stress and Health, PLA Strategic Support Force Medical Center, Beijing, P. R. China; Department of Otorhinolaryngology Head and Neck Surgery, PLA Strategic Support Force Medical Center, Beijing, P. R. China; Department of Gastroenterology, PLA Strategic Support Force Medical Center, Beijing, P. R. China

**Keywords:** organoids, pancreatic ductal adenocarcinoma, ultrasound-guided fine-needle aspiration/biopsy, review

## Abstract

Pancreatic ductal adenocarcinoma (PDAC), as one of the malignant cancers with the worst prognosis, is becoming the most urgent clinical problem. Due to the lack of early diagnosis and curable therapeutic methods, it is critical to exploit proper models that can capture the overall attributes of the primary tumor. Recently, organoid technology has emerged and flourished as a powerful tool to enable long-term culture of pancreatic tissues, including PDAC. As accumulating studies suggest, organoids can retain morphological, genetic, and behavioral traits, and have tremendous value in predicting the therapeutic response to conventional chemotherapy drugs or newfangled agents. Herein, this review comprehensively summarizes the tissue source including human fetal and adult pancreatic tissue to generate a pancreatic organoid as well as current organoids cultivate system. As PDAC organoids can be established from a small number of samples derived from endoscopic ultrasound-guided fine-needle aspiration/biopsy (EUS-FNA/FNB), we also review the literature to date on EUS-FNA/FNB-based organoid constitution and its implementation in inquiring tumor behavior and evaluating therapeutic responses. By enabling the alignment of basic and clinical research platforms, the application of organoids would open up new avenues for drug discovery and maximally benefit translational medicine in the near future.

## Introduction

Three-dimensional primary tissue culture, also termed as “organoids,” is defined as a reconstruction of the tissue or organ in a 3D structure derived from primary cells or pluripotent embryonic or adult mammalian stem cells, which preserve the genetic and phenotypic attributes and resemble the functionality of original organs [1]. The evolution of organoids mainly originated from Clevers laboratory in 2009 [2]. They sorted lgr5^+^ stem-like cells from the intestinal epithelium, which successfully evolve into the self-organizing structures without mesenchymal cellular niche. The area of application of organoid technology in the digestive system consists of the esophagus [3], stomach [4], liver [5], bile duct [6], pancreas [7], intestine [8], and their corresponding cancer tissues. Previously, organoid culture methods were mainly applied to the culture of normal tissues. With the deepening of research regarding the origin and self-repair of the pancreas, the deficiency of Wnt signaling and lgr5^+^ pancreatic cells appears in the healthy and intact pancreas [9]. As the integrity of the pancreas is impaired, Wnt and lgr5^+^ cells are robustly activated and upregulated, particularly in pancreatic ducts. As the combination of organ regeneration with the advancement of pancreatic culture technology exhibits great application potential, organoids of tumors or other disease types are gradually being established and evolved. Since the first research on the establishment of organoids from normal and pancreatic ductal adenocarcinoma (PDAC) tissues of human and mouse was successfully reported in 2015 [10], organoids, accompanied by their attributes of intactly recapitulating the landscape of the genome and transcriptome and ready genetic manipulation, have been emerging as a burgeoning and facile model for mimicking the developmental phase and testing therapeutic options for pancreatic diseases [11].

PDAC, like other malignant tumors, retains remarkable inter-tumoral and intra-patient heterogeneity of phenotype and molecular profiles [12]. In the past, patients with advanced or unresectable PDAC were given up on for the reason that we lacked the approaches for obtaining a sufficient number of tumor tissues for further studies [13]. To address this problem, the appearance of the endoscopic ultrasound-guided fine-needle aspiration/biopsy (EUS-FNA/FNB)-based patient-derived tumor xenograft (PDTX) has provided another better option for predicting the response to certain chemotherapy regimens [14]. However, the time consumption and dissatisfactory success rate of constructing a maturely individual PDTX model have prevented it from systematic application in clinics due to a short lifetime for patients with advanced PDAC. Compared with PDTX, EUS-FNA/FNB-derived patient-derived organoids (PDOs) culture is easily manipulated and has a higher success rate of 70%–80% [15]. Therefore, PDOs are now regarded as the most promising tool for evaluating the molecular signature and chemosensitivity in a personalized manner in PDAC patients.

In the present review, we will discuss the tissue sources and methods of organoid-like culture, and then review the current knowledge about the role that EUS-FNA/FNB plays in obtaining tissues for culturing PDOs and subsequently analysing molecular characteristics.

## Methodology of pancreas organoids culture

The adult human pancreas mainly comprises three cell types: ductal, acini and endocrine (islets) cells. Conventional monolayer cell culture is believed to have laid the cornerstone of modern cell biology. Nevertheless, it is regrettable that not all pancreatic tissues have corresponding cell lines [16]. Furthermore, primary culture of pancreatic tissue in the culture dish is not an easily manipulable task, whether it is attached to the bottom or suspended in media. Primary cells in the dish will gradually lose the originally differentiated state or cease to expand. Some studies have also reviewed the possible reasons for the above situation [17, 18]. To make matters worse, the results of cell culture-based drug exploitation and verification are, to a large extent, inconsistent with clinical trials [19, 20]. High-throughput screen techniques such as next-generation sequencing have confirmed that there are many differences in expression genomics and transcriptome between traditional cell culture and primary lesions [21–23]. Poor representation and methodological imperfections of the monolayer 2D cell culture are becoming an obstacle to basic research and therapeutic applications.

## Organoids from embryonic pancreas or human pluripotent stem cells

To decipher the pathophysiological process of pancreas development and diseases better, the emergence of the organoid culture provides another alternative to disentangle the sophisticated mechanisms. Modern pancreatic organoids that often employ Matrigel as the scaffold are cultured in a well-defined medium [24]. Previous study has indicated that cells that express SRY-box transcription factor 9 (SOX9) in the mouse fetal pancreas have the capacity to regenerate endocrine and acinar cells. However, there is no neogenesis of endocrine or acinar from SOX9^+^ cells during adulthood [25]. Diverse studies make use of different cell markers, which represent pluripotent stem cells (PSCs), to isolate cells that can differentiate into organoids from the pancreas and their cultivation methods are also slightly different. In 2013, a study [26] reported that three unique culture systems for isolating and expanding the progenitor cells into spheres from fetal mouse pancreas were developed. They first sorted out Sox9-eGFP^+^ Ngn3-tdT^neg^ cells by fluorescence-activated cell sorting and then plated diluted cells into the Matrigel submerged in the basal PrEBM medium added with insulin, transferrin, mouse insulin-like growth factor 1, and bovine pituitary extract. The sorted spheres were demonstrated to express progenitor markers including SOX9, pancreatic and duodenal homeobox 1 (PDX1), and hepatocyte nuclear factor 6. To expand the sphere, the basal medium was supplemented with human fibroblast growth factor 10 (FGF10), B-27 supplement, and all-trans retinoic acid, and cultured under an oxygen-enriched environment (21% O_2_). When the basal medium was added with FGF10 and nicotinamide and cultured under the normal oxygen level (5% O_2_), the spheres could differentiate and possess the cardinal characteristics of α-cells and β-cells. As for the studies of the human fetal pancreas, the scarcity of human embryonic tissues and ethical restrictions give rise to insufficiency of comprehensive knowledge of the human pancreas and development [27, 28]. Bonfanti *et al.* [29] employed human fetal pancreas as well as mouse fetal pancreas to generate cells that can expand for many passages and be induced into a hollow duct structure, namely organoids in the medium with Wnt agonist R-Spondin1, FGF10, and epidermal growth factor (EGF). These fetal organoids also express progenitor markers such as PDX1, NK6 homeobox 1, and SOX9. However, in the absence of EGF, the ability of proliferation was suppressed while the differentiation into endocrine cells was initiated. In conclusion, EGF-signaling actively participated in the modulation of the expansion and differentiation of the organoids from fetal human and mouse pancreases.

Another route for obtaining pancreas progenitor cells that can develop into organoids is PSCs lines. A study demonstrated that by adding EGF and nicotinamide signaling inductive substance in 4 stage of culture, pancreatic endoderm can be established from PSCs [30]. Along this line, amplification and induction of PSCs that contain a NKX6.1^+^PDX1^+^ progenitors subpopulation to the mature pancreatic organoid *in vivo* are divided into two phases: in the first 8 days of culture, the PSCs were plated in 3D culture with the medium including B-27, FGF2, insulin, hydrocortisone, and ascorbic acid; from the 9th day, the medium was changed into B-27 serum-free medium supplemented with ascorbic acid and the cells were cultured for another 8 days. After 16 days of culture, the PSCs lines evolved into organoids with polarized 3D structures. The levels of expression of pancreas-specific markers including NKX6.1 and pancreas-associated transcription factor 1a were markedly elevated in organoids. By modifying the compounds in the medium in the second phase, the differentiation of the progenitor organoids into CA2^+^ ductal or CPA1^+^ acinar population can be implemented [31].

No matter whether progenitor cells are isolated from the embryonic pancreas or human embryonic stem cell induced pancreas progenitor *in vitro*, efforts are needed to manipulate cardinal developmental signaling pathways such as Wnt and Hedgehog pathways precisely. The differentiated fates of organoids to the endocrine cells or exocrine cells *in vivo* or *in vitro* are complicated by the two programs [9, 32]. Although the regulatory mechanisms are intricate, with the improved genetic screens and synthetic drug induction, this system will scale up to clarify the process of pancreatogenesis and development [30, 33].

## Organoids from adult pancreas

The absence of progenitor cells or PSCs in adult pancreas, compared with the fetal pancreas, accounts for the primary reason why there are various discrepancies such as morphology and endocrine cellular differentiation between organoids from fetal and adult pancreases [10, 34, 35]. In 2013, the study from Huch *et al.* [9] demonstrated that upon partial pancreatic duct ligation being conducted in mice, Wnt signaling as well as Lgr5^+^ stem-like cells were initiated. Both pancreatic duct fragments and sorted single cells that contain Lgr5^+^ cells can grow and form into organoids that are embedded in Matrigel with the medium containing EGF, R-Spondin1, Noggin, FGF10, and nicotinamide. Long-term culture for organoids over eight passages and successful transplantation and formation of tumor were constituted in the immunodeficient mice. Subsequent research also revealed that organoids that have originated from not only Lgr5^+^ cells but also adult duct fragments acquire the bio-potency of differentiating ducts and endocrine cells [10].

Apart from the tissues or sorted cells from pancreatic ducts, centroacinar/terminal ducts cells (CA/TD) that are reported to be located at the junction between peripheral acinar cells and the adjacent ductal epithelium also take part in the resource of organoids. The author found that CA/TD expressed aldehyde dehydrogenase 1 enzymatic activity at a higher level, which can be used to sort and isolate by using fluorescence-activated cell sorting. Despite the depletion in the ability to differentiate to mature pancreatic cell types, some progenitor markers in embryonic pancreas were greatly enhanced. Suspended CA/TD in culture with the presence of EGF, FGF2, leukemia inhibitory factor, and serum can spontaneously form compact “pancreatospheres,” full of cells, namely pancreatic organoids. Progressively, pancreatospheres displayed certain functions of mature pancreas such as secreting insulin in response to high glucose [36]. Some studies also noted that the dispersed cells from islet or whole pancreas can be cultured and differentiate into endocrine cells in suspension with FGF2, Heparin, EGF, serum, and glucagon-like peptide-1 [37]. An intriguing study demonstrated that both mouse and human pancreas-derived multipotent precursor that was isolated by lineage-labeling experiments possessed multiple traits such as proliferation, self-renewal, and differentiation [38]. This experiment was also the predictor of the diversity of the source of pancreatic organoids.

## EUS-FNA/FNB as a route for obtaining material and generating PDOs of PDAC

The combination of an ultrasound transducer with an endoscope came forth in the early 1980s [39]. Yet, it was not globally put into clinical use until ultrasonic guided biopsy needle during the examination procedure occurred [40, 41]. Over the years, EUS-guided tissue sampling, namely EUS-FNA/FNB, has been widely adopted to detect and differentially diagnose pancreatic lesions, including PDAC. Obviously, EUS-FNA/FNB has been marked as the milestone since it has been proven to be a safe procedure [42]. PDTX models, in the past, were considered as a personalized tool for cancer treatment. It is disappointing that the number of patients whose resectable surgical specimens can be used to construct PDTX is quite limited, ranging from 10% to 15% [43]. Based on EUS-FNA/FNB techniques, Allaway *et al.* [44] successfully established PDTX models from the primary site as well as two metastatic sites of PDAC from one patient. They also verified the role of cyclin-dependent kinase inhibitors in inhibiting tumor proliferation and inducing tumor cell apoptosis in FNA-PDTX and patient-matched metastatic-PDTX models. There is no doubt that EUS-FNA/FNB-based PDTX provides a novel approach for predicting antitumor drug response precisely. Nevertheless, PDTX is limited in its clinical application due to its own defects such as being time/money-consuming and having a low engraftment success rate [45].

**Figure 1.**
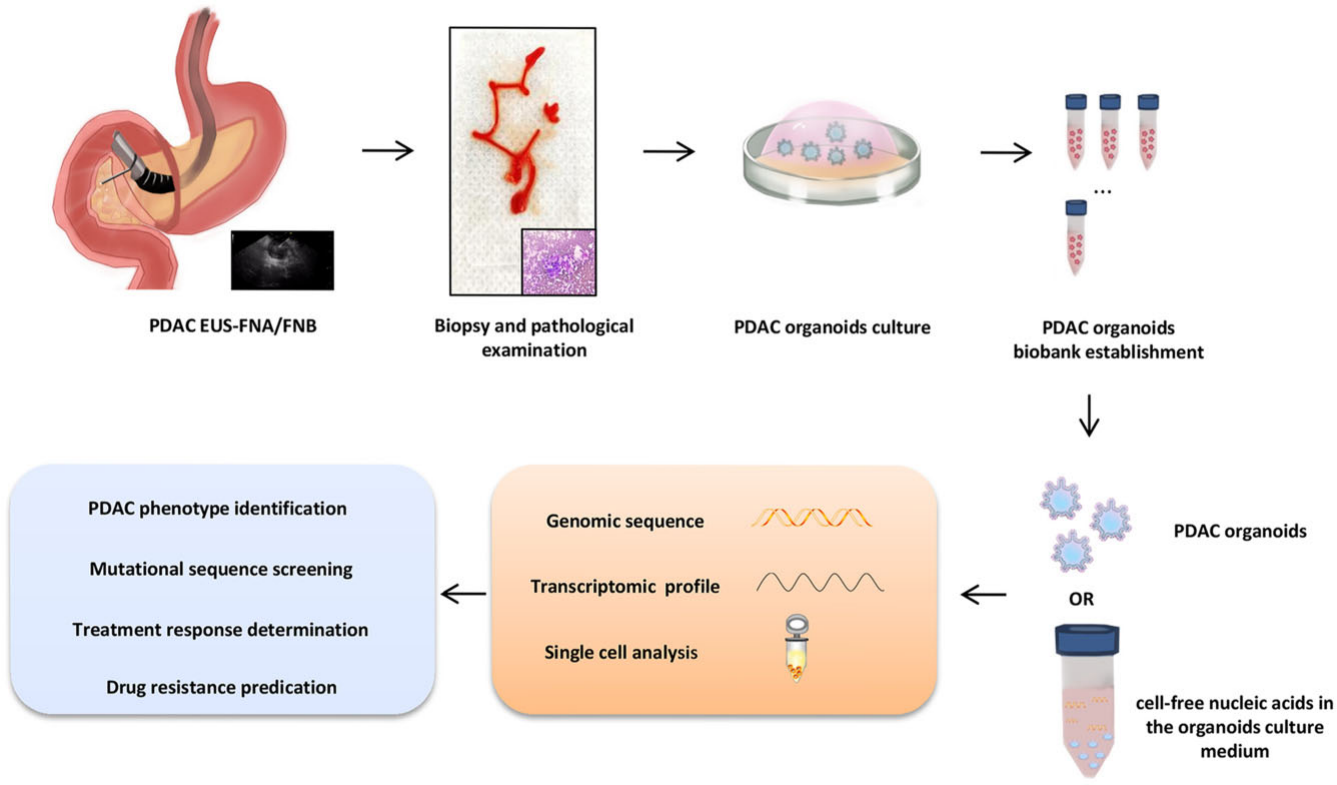
The schematic flow chart of basic and preclinical application of PDAC organoids. First, the PDAC biopsy samples and subsequent pathological examination can be conducted in most non-operable PDAC patients through EUS-FNA/FNB. Next, the PDAC biopsy samples are purified and incised. The prepared tissues are cultured in Matrigel as the scaffold and PDAC PDOs can replicate in a well-defined selective medium. After 2–3 weeks, adequate organoids can be obtained and stored for the construction of PDOs biobanks. The abundant genetic information in PDOs and even in the culture medium can, to the maximum extent, mimic the tumor microenvironment and be detected through various molecular biological techniques. In the near future, PDOs can not only act as a robust tool for predicting the prognosis of PDAC patients, but also provide individualized and appropriate therapy. PDAC, pancreatic ductal adenocarcinoma; EUS-FNA/FNB, endoscopic ultrasound-guided fine-needle aspiration/biopsy; PDOs, patient-derived organoids.

To figure out these puzzles, scientists have utilized EUS-FNA/FNB to obtain a small number of biopsy specimens to cultivate high-purified PDAC organoids and then applied them to probe the genetic characteristics of primary tumors and individualized therapy in PDAC patients owing to the relatively easier establishment and higher success rate of PDOs (Figure 1). In 2018, Herve Tiriac and his colleagues reported that they managed to isolate and establish 33 PDAC organoids in 38 PDAC tumors (P0) within 2 weeks through EUS-FNB with 22G biopsy needles [15]. Moreover, 66% of organoid lines passed over five cycles of growth (P5). In 2020, the same group compared the success rate of organoids establishment in a single pass with two-pass FNB. The result showed that the biopsy pass number had no impact on the P0 establishment and P5 proliferation rate, which proved that one needle pass seemed safe and feasible [46].

Other work from Natalia Juiz *et al.* [47] revealed that almost every EUS-FNA-derived classical PDAC organoid was subjected to single-cell transcriptomics analysis and then presented four main cell clusters in different proportions. It is beyond expected that one of the four cell clusters belongs to a basal-like phenotype, which is highly aggressive. As for the antitumor drugs test and screen, organoids from EUS-FNA/FNB display their unique advantages in a rapid and high-throughput manner. One study in 2019 demonstrated that EUS-FNA-derived PDAC organoids conserve the same attributes as the primary tissue such as morphology and the signature of the MYC gene [48]. On the basis of the MYC expression level in organoids, they divided organoids into MYC-high and MYC-low subgroups. JQ1 and NHWD-870, both of which are selective BET family bromodomain inhibitors, are more efficient in MYC-high organoids. Another elegant study regarding chemotherapy selection in PDAC took advantage of PDOs to nominate the gene expression signature that can predict clinical response to conventional chemotherapy [49]. They first generated a library of 159 organoids, 60 of which were from EUS-FNA/FNB biopsy. Following whole-exome sequencing and transcriptomic profiling, organoids were classified as two distinct clusters, C1 and C2, according to unique gene expression programs. Then, 66 organoids underwent a “pharmacotyping” process and displayed distinct sensitivity to four common PDAC chemotherapeutic agents. For one patient, longitudinal repeat EUS-FNA-derived PDOs demonstrated changes in drug resistance as the PDAC progressed. In the end, for chemorefractory PDOs, alternative therapies were introduced. More recently, instead of focusing on detecting PDAC PDOs themselves, an intriguing article reported that authors collected the conditioned medium of PDAC PDOs and tested the cell-free DNA in the medium [50]. As early as 72 hours after biopsy, cell-free DNA in the organoids medium could recapitulate driver mutations in the primary tumor before the expansion of organoids. This discovery can further reduce the drug-testing time-frame consumption, especially being practicable in patients whose original tumor sampling quantity is not sufficient for PDOs culture and subsequent molecular characterization. With this innovative approach, rapid and accurate acquisition of therapy testing and gene profiling could be achieved with multiple possibilities.

EUS-FNA/FNB-derived pancreatic PDOs have been proven to be an efficient approach for establishing a PDO biobank. Some studies compared the features of EUS-FNA/FNB PDAC organoids with the organoids from surgical resection. A study from Seppälä in 2020 reported that PDAC organoids that are established from endoscopic and surgical biopsies share almost the same success rate [51]. In 2022, in order to evaluate the response to neoadjuvant chemotherapy, a study implemented by Demyan *et al.* [52] recruited 94 patients with confirmed PDAC. Among the patients, the successful rate of EUS-FNA- and FNB-derived organoids was 56% and 53%, respectively, while the rate of surgical specimens from chemotherapy-naive tumors and chemotherapy-treated tumors is 76% and 71%, respectively. It seemed that surgically derived organoids are more likely to survive and passage due to the additional number of cells in the resected bulk. However, as the construction of a biobank of organoids is generally the first step to evaluate the applicability of organoids, EUS-FNA/FNB and surgery perform different roles in acquiring organoids in different treatment phases. Surgically derived organoids can be acquired for patients with early pancreatic cancer or patients undergoing neoadjuvant chemotherapy. Nevertheless, during pre-chemotherapy and post-chemotherapy stages, FNA/FNB can act as an active role in acquiring biopsy to establish and compare the biological hallmarks of PDAC organoids. The estimation of features of organoids can help clinicians with late-treatment decision-making [53–55].

## Clinical applications of organoids in pancreatic diseases

Since PDAC harbors inter- and intra-tumor heterogeneity, antitumor therapies, especially chemotherapeutic regimens, lack effectiveness and induce drug resistance. Surgical resection or EUS-FNA/FNB-based PDAC organoids precision oncology platform establishment exhibits tremendous potential to be a powerful preclinical tool to predict outcomes of pharmaceutical chemotherapy. In 2022, Demyan reported that the response of PDAC organoids to chemotherapy, especially oxaliplatin, can predict the response of matched patients [52]. In the same year, a study from Peschke constructed a longitudinal framework of PDAC therapeutic effect from a diagnosed-confirmed PDAC patient [53]. EUS-FNB biopsy-derived PDOs were obtained before receiving neoadjuvant treatment and another set of PDOs were also generated from surgical specimens after chemotherapy induction. After RNA-sequence and pharmacotyping analysis, metabolic pathways and conventional chemotherapy drug responses altered massively. Another study from Hogenson also demonstrated that PDAC organoids, either from EUS-FNB biopsy or surgical resections, could be used to predict the drug response and harness personalized treatment [54]. What can really make a difference in the efficacy of drug prediction is the composition of the organoids cultural media.

As pioneered for PDAC, pancreatic organoids would have versatile traits in other pancreatic diseases, especially some benign ones. Diabetes, either type 1 or type 2, is prevalent. Both genetic defects and environmental factors result in complicated etiology. Transplantation of pancreas or isolated pancreatic islets can bridge the cell-based cure in the laboratory with clinical applications. Organoids, compared with traditional PSC-derived β cells, conserve the crosstalk between endocrine cells and other types of cells such as hormone-producing and acinar cells. The crosstalk helps to leverage the differentiation rate of organoids into insulin positively functional cells in a dish [56]. Some animal research also showed that by using certain markers, sorted cells from murine islets can generate organoids that contain all kinds of islet cells [57]. In general, the research of organoids in benign pancreatic diseases is not yet as generally prevalent clinically as PDAC. But organoids remain a tool for investigating the mechanism of insulin-producing islet cells.

## Conclusions

Human pancreatic organoids are constructing a bridge between basic and preclinical therapy research and have resulted in unprecedented advances in the study of PDAC over the past decade. Hence, organoid technology was crowned as the “Method of the Year” by *Nature Methods* in 2017 [58]. In this review, we discussed the current knowledge of the source of pancreatic organoids generation. Although the exact section of fetal or adult pancreas that can expand and differentiate into organoid remains vague, the increasing understanding of the mechanism of development, differentiation, and homeostasis of the pancreas will shed light upon the procedure of organoid regeneration. Additionally, we explored the role and application development prospects of EUS-FNA/FNB in the establishment of organoids from PDAC patients. Until now, the best way to obtain information on non-resectable and metastatic PDAC has been EUS-FNA/FNB PDOs. Nevertheless, this technology still has some weaknesses. On the one hand, stromal or blood cells in the EUS-FNA/FNB biopsy sometimes proliferate faster than the tumor epithelial cells, thus they suppress the formation of PDAC organoids and contaminate the following sequence results. On the other hand, RNA degradation caused by RNAses in the biopsy samples still remains a non-negligible problem for the next genome and transcriptome analysis [59]. Moreover, on account of the high purity of PDAC in organoids culture, the absence of the tumor microenvironment components such as immune and stromal intercrossing may disable organoids in recapitulating the complexity of the tumor.

In summary, though extensive optimization for the current organoids establishment framework is needed, it is definitively imperative to establish the corresponding PDAC organoid model in the early stage of diagnosis. The PDOs platforms bring the basic research one step closer from the bench to clinical practice. The era of “organoidomics” [60] is just around the corner and we would envision that they will impose a substantial clinical impact in the near future.

## Authors’ Contributions

W.W. and Lianyong Li provided the idea for the review and revised the manuscript. C.Z. and M.W. performed the literature search. S.C., L.W., G.W., C.G., and Lei Liu conducted the data analysis and drafted the manuscript. All authors read and approved the final version of the manuscript.
